# T Cell Response following Anti-COVID-19 BNT162b2 Vaccination Is Maintained against the SARS-CoV-2 Omicron B.1.1.529 Variant of Concern

**DOI:** 10.3390/v14020347

**Published:** 2022-02-08

**Authors:** Hila Cohen, Shahar Rotem, Uri Elia, Gal Bilinsky, Itzchak Levy, Theodor Chitlaru, Erez Bar-Haim

**Affiliations:** 1 Department of Biochemistry and Molecular Genetics, Israel Institute for Biological Research, Ness-Ziona 74100, Israel; hilac@iibr.gov.il (H.C.); shaharr@iibr.gov.il (S.R.); urie@iibr.gov.il (U.E.); galb@iibr.gov.il (G.B.); theodorc@iibr.gov.il (T.C.); 2 Sheba Medical Center, Infectious Disease Unit, Ramat Gan 5262112, Israel; Itsik.Levy@sheba.health.gov.il; 3 Sackler Medical School, Tel Aviv University, Tel Aviv 6997801, Israel

**Keywords:** SARS-CoV-2, COVID-19, Omicron, T cell responses, ELISPOT, FluoroSpot, variants of concern

## Abstract

The progression of the COVID-19 pandemic has led to the emergence of variants of concern (VOC), which may compromise the efficacy of the currently administered vaccines. Antigenic drift can potentially bring about reduced protective T cell immunity and, consequently, more severe disease manifestations. To assess this possibility, the T cell responses to the wild-type Wuhan-1 SARS-CoV-2 ancestral spike protein and the Omicron B.1.1.529 spike protein were compared. Accordingly, peripheral blood mononuclear cells (PBMC) were collected from eight healthy volunteers 4–5 months following a third vaccination with BNT162b2, and stimulated with overlapping peptide libraries representing the spike of either the ancestral or the Omicron SARS-CoV-2 virus variants. Quantification of the specific T cells was carried out by a fluorescent ELISPOT assay, monitoring cells secreting interferon-gamma (IFNg), interleukin-10 (IL-10) and interleukin-4 (IL-4). For all the examined individuals, comparable levels of reactivity to both forms of spike protein were determined. In addition, a dominant Th1 response was observed, manifested mainly by IFNg-secreting cells and only limited numbers of IL-10- and IL-4-secreting cells. The data demonstrate stable T cell activity in response to the emerging Omicron variant in the tested individuals; therefore, the protective immunity to the variant following BNT162b2 vaccination is not significantly affected.

## 1. Introduction

Omicron B.1.1.529 is currently the prevalent variant of concern (VOC) amongst the emerging SARS-CoV-2 variants [1]. The Omicron variant was first described in November 2021 and, since then, has been rapidly spreading worldwide [1,2]. It bears 26–32 mutations in the spike protein compared with the Wuhan-1 SARS-CoV-2 sequence [1], many of these mutations being located in the receptor binding domain (RBD). As was shown for other VOCs [3], mutations in the neutralizing sites of the spike protein weaken the neutralizing potential of antibodies and, consequently, may lead to enhanced immunological escape. This aspect has tremendous public health implications, considering the massive on-going vaccination campaigns worldwide based on the antigenic specificity of the primordial SARS-CoV-2 strain.

As of today, precise correlates of protection against SARS-CoV-2 have not been fully defined. It is clear that the neutralizing antibody response is essential for blocking viral attachment and entry to host cells, and that T cells play a central role in diminishing viral spread in the host, thus alleviating the severity of disease manifestation [4]. Accordingly, for several emerging VOCs, it was shown that a lower neutralizing antibody response was correlated with lower efficiency of the vaccine and higher levels of immune breakthrough infections [4,5]. Considering the antibody titer kinetics following vaccination and their potential waning below the neutralizing levels, it is essential to maintain protective T cell memory responses, which are expected to exhibit significant longevity [6,7,8]. Immune escape from the humoral response is mostly a result of specific mutations of a given antigen, which occur in a convergent microevolutionary process and therefore affect equally different individuals. Conversely, the T cell response has a divergent character, distinctly affecting various individuals due to HLA polymorphism; therefore, unique mutations in the immunodominant epitopes are less likely to affect the T cell responses globally. Weakening of T cell immunity against a VOC may occur as a consequence of antigenic drift that leads to accumulated mutations underlying immunity [9].

T cell responses may provide protection from SARS-CoV-2 even in the absence of an antibody response [10]. Specifically, high levels of IFNg-secreting cells responsive to antigenic stimulation with the SARS-CoV-2 spike protein correlated with less severe COVID-19 disease manifestations [10]. Monitoring the T cell response is experimentally more challenging than quantification of the humoral responses, requiring the availability of viable cells responding to antigen stimulation. Most studies characterized T cell responses by activation-induced marker (AIM) elevation and cytokine intracellular staining, monitored by flow cytometry and by ELISPOT assays [11]. In the current study, we determined the level of T cell reactivity in response to the ancestral Wuhan-1 SARS-CoV-2 spike and the Omicron B.1.1.529 variant spike in healthy individuals immunized with the BNT162b2 vaccine. The study revealed a similar, dominant Th1 response to both versions of the spike protein, suggesting that stable T cell immunity is maintained against the currently prevalent Omicron variant.

## 2. Materials and Methods

### 2.1. PBMC Isolation

Blood was collected from 8 healthy individuals within 4–5 months of their third BNT162b2 vaccine. Blood was collected into sodium-heparin tubes (vacutainer, BD, Franklin Lakes, NJ, USA) and processed within 2 h of collection. Peripheral blood mononuclear cells (PBMC) were isolated by density gradient sedimentation using Ficoll-Paque (Sigma-Aldrich, Rehovot, Israel) according to the manufacturer’s protocol. Cells were then washed once in PBS and immediately processed for ELISPOT assay.

### 2.2. ELISPOT Assay

The three-color fluorescent ELISPOT assay (FluoroSpot) was performed with strict adherence to the manufacturer’s protocol (Human IFN-γ/IL-4/IL-10 Three-Color FluoroSpot, ImmunoSpot, Cleveland, OH, USA). PBMC were resuspended in FCS-free CTL-Test media (ImmunoSpot) and plated in 96-well PVDF membrane plates at 3 × 105 cells/well. Cells were either left unstimulated, stimulated with SARS-CoV-2 spike protein overlapping the peptide library or stimulated with 5µg/mL of phytohemagglutinin (PHA) (Sigma-Aldrich, Rehovot, Israel) as a positive control. Commercially available peptide pools (15-mer sequences with an overlap of 11 amino acids) covering the full length of the Wuhan-1 SARS-CoV-2 (wild-type) or Omicron B.1.1.529 variant spike (peptides & elephants GmbH, Hennigsdorf, Germany) were used for PBMC stimulation. Peptide pools were dissolved in DMSO and used in a final concentration of 200 µg/mL (0.6 µg/mL per peptide); DMSO’s final concentration was below 0.1%. The plate layout is presented in Supplementary Figure S1. PBMC were stimulated for 48 h, and the frequency of cytokine-secreting cells was quantified with the ImmunoSpot S6 Ultimate reader with the 520, 600 and 690 nm filters to allow enumeration of cells expressing IFNg, IL-10 and IL-4, respectively. Data were analyzed with ImmunoSpot software version 7.0.30.2 (ImmunoSpot). Positive spots overlapped by the 520 nm and 600 or 690 nm filters were considered as potential artefacts and excluded. Statistical significance analysis of the data was performed by Student’s *t*-test.

## 3. Results and Discussion

Cellular immunity is instrumental in preventing severe COVID-19. In the current study, which included eight healthy individuals (referred to as donors), vaccinated in Israel three times with the mRNA BNT162b2 vaccine, we sought to determine and compare the level and type of T cell response to either the Wuhan-1 SARS-CoV-2 spike or the Omicron B.1.1.529 variant. The donors were 20–52 years old (average 27.1,) and included three males and five females. No COVID-19 history was documented for any of the donors; furthermore, considering the accurate epidemiologic registration customary in Israel, it is inconceivable that they were previously infected.

**Figure 1:**
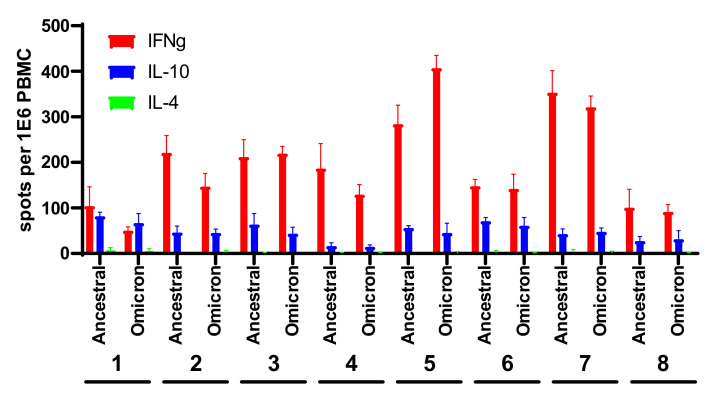
T cell response to the ancestral and Omicron SARS-CoV-2 spikes in BNT162b2-vaccinated individuals. PBMCs were stimulated with ancestral or Omicron spike-derived overlapping peptides. IFNg-, IL-10- and IL-4-secreting cells were quantified in a FluoroSpot assay. Data represent the average and standard deviation of four replications of each experimental group. Numbers 1–8 refer to 8 different donors from which PBMCs were collected.

**Figure 2:**
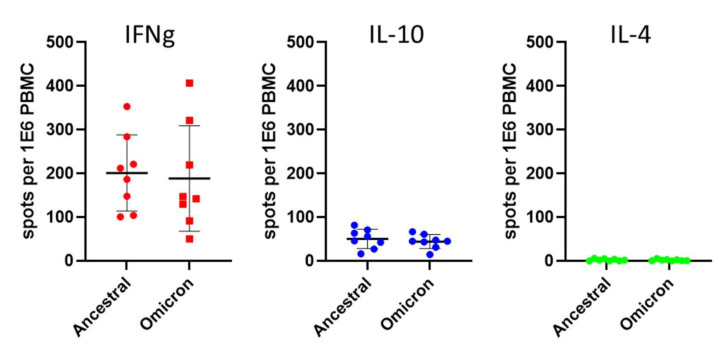
Comparative analysis of the T cell response to ancestral and Omicron spike protein. PBMCs were stimulated as described in Figure 1. Each dot represents the average of four measurements of the same sample. Averaged and standard deviationd of the data from the independent donors for each cytokine and antigen are presented. The colors employed for the three different cytokines are as detailed in Figure 1.

PBMC collected from the donors were stimulated with a mixture of 315 peptides, each 15 amino acids long, spanning the entire spike protein. The study inspected both the type and the level of response, as determined by the number of cytokine-secreting cells in a fluorescent ELISPOT assay. Following antigenic stimulation with the spike-derived peptides, a predominant IFNg response was observed in all the examined individuals, ranging from 50 to 400 secreting cells per 10^6^ PBMC, and a lower IL-10 response, ranging from 15 to 82 cells per 10^6^ PBMC (Figure 1 and Figure S1). The experimental setup included a 48 h antigen stimulation step to allow manifestation of the reactivity of IL-4-expressing T cell clones potentially present in the samples; however, almost undetectable levels of IL-4 reactivity were determined, in accordance with previous data pertaining to the responses elicited by the BNT162b2 and m1273 vaccines [2,4,8,9,10,11,12,13]. A comparison of the average response to the wild-type and Omicron spike (Figure 2) indicated only a slight non-significant decrease from 201 IFNg-secreting cells following activation with the wild-type spike, to 188 cells responding to the Omicron spike. Furthermore, no significant differences were observed between the responses measured on the basis of IL-10- and IL-4-secreting cells. With respect to the IFNg induction of each tested individual (Figure 1), in one case (Donor 2), statistically significant preferential activation by ancestral spike peptides was observed, yet in another case (Donor 5), preferential activation was exhibited in response to the Omicron spike. Of note, no significant differences were recorded for the IL-10- and IL-4-secreting cell quantifications. The IFNg response was higher than that of IL-10, with the average ratio of IFNg/IL-10 response being 4.9, indicating a dominant Th1 response with no significant Th2 response.

The essentiality of T cells for protection against COVID-19 is well documented [2,4,10]; therefore, confirmation of T cell reactivity towards emerging VOCs is of outmost importance. As newer emerging VOCs are identified, maintenance of the long-term protective immunity of vaccinated individuals represents a public health concern of high priority. It was suggested that in the case of several VOCs, convalescent and vaccinated individuals exhibited some escape from humoral immunity, while revealing normal uncompromised T cell reactivity [5,14].

In the present study, by analyzing the response in individuals following three BNT162b2vaccines, we showed a dominant Th1 response to the Omicron variant spike protein, which correlates with protective immunity [6]. The T cell responses to both the ancestral and Omicron variants were of commensurate levels (Figure 2), in line with several recent reports [2,12,13]. Since our data show comparable levels of response to both ancestral and Omicron spikes, it is reasonable to estimate that the CD4 and CD8 composition of the T cell compartment remains steady for the response to both variants. The added value of a third vaccination for achieving Omicron antibody neutralization was previously demonstrated [15], and future studies will address the relevance of third vaccine administration in maintenance of the T cells’ responses as well. In addition, additional studies are expected to monitor other population fractions, especially older people at a high risk of developing severe forms of COVID-19.

## Data Availability

Not applicable.

## Supplementary Materials

The following supporting information are available at https://www.mdpi.com/article/10.3390/v14020347/s1. Figure S1: FluoroSpot plate layout and quantitation.
